# Antimicrobial Activity of Carboxymethyl Cellulose Films Containing Plantaricin W and Enterocin F4-9 for Meat Preservation

**DOI:** 10.3390/ijms26136083

**Published:** 2025-06-25

**Authors:** Mohamed Abdelfattah Maky, Kenji Sonomoto, Takeshi Zendo

**Affiliations:** 1 Department of Bioscience and Biotechnology, Faculty of Agriculture, Graduate School, Kyushu University, 744 Motooka, Nishi-ku, Fukuoka 819-0395, Japan; mohamedmekky@vet.svu.edu.eg (M.A.M.); sonomoto@kyudai.jp (K.S.); 2 Department of Food Hygiene and Control, Faculty of Veterinary Medicine, South Valley University, Qena 83523, Egypt

**Keywords:** food packaging, plantaricin W, enterocin F4-9, minced meat

## Abstract

Antimicrobial food packaging is considered a promising technology to improve food safety by inhibiting or reducing the growth of food microorganisms and minimizing the need for preservatives. This study aimed to develop and evaluate carboxymethyl cellulose (CMC) films integrated with bacteriocins for antibacterial efficacy. Plantaricin W was assessed as a potential bacteriocin for activation of CMC to control the dangerous food-borne pathogen, *Listeria monocytogenes*. Minced beef samples were inoculated with *L. monocytogenes* ATCC BAA-679 and treated with plantaricin W-activated food packaging. The results showed a significant reduction of the target pathogen by approximately 1 log cycle compared to the control group. Enterocin F4-9 is a novel bacteriocin that acts on Gram-negative microbes that were not affected by plantaricin W. Therefore, a novel food packaging activated with plantaricin W and enterocin F4-9 was developed to broaden their antimicrobial activity. The effect of this film on meat-associated microbes was investigated. The results demonstrated that the film significantly reduced the counts of mesophilic and psychotropic bacteria by 86.67% and 96.67%, respectively. Additionally, the pH values of the treated meat samples were significantly lower than those of the untreated controls. The obtained findings indicated that bacteriocin-activated CMC films could potentially be utilized as antimicrobial packaging in modern food technology.

## 1. Introduction

Control of pathogenic and spoilage bacteria is an important aspect of food safety. Effective methods should be used to maintain the physical, chemical, and microbiological properties of foods [1]. *Listeria monocytogenes* is a widespread Gram-positive bacterium in the environment. It can cause listeriosis, a fatal invasive illness in humans. It is extremely fatal in persons who have impaired immune systems, pregnant women, and children. *Listeria monocytogenes* is one of the harmful food-borne pathogens, which has been isolated from various food items such as raw and processed meat, milk, and seafood [2]. Many groups of mesophilic and psychrotrophic aerobic bacteria participate in meat spoilage even under appropriate preserving conditions and lead to changes in the chemical properties of meat such as increasing the pH of meat. Many approaches have been developed to control these biohazards in meat, but the consumer demands for more natural food have encouraged food scientists to discover a novel method for preservation using natural compounds: biopreservation. Biopreservation means prolonging the shelf life and maintaining the safety of food using microorganisms and/or their metabolites, so-called biopreservatives [3]. Bacteriocins are one type of biopreservative as they follow the general features of biopreservative materials [4].

Bacteriocins are defined as antimicrobial peptides ribosomally synthesized by prokaryotes, which show antibacterial activity against species closely related to the producer strain [5,6]. Bacteriocins produced by lactic acid bacteria (LAB), which are generally recognized as safe microorganisms, have received considerable attention in food preservation. LAB bacteriocins have various desirable features, including nontoxicity, easy degradation by human proteases, and antimicrobial activity against many Gram-positive food-borne pathogens and spoilage bacteria. These features have encouraged food researchers to evaluate the potential application of these preservatives as promising biopreservatives.

The incorporation of bacteriocins into food packaging is one of the widely used methods for the application of bacteriocins in food [7]. Antimicrobial food packaging can kill or inhibit spoilage and pathogenic microorganisms to meet consumer demands for high-quality and safe food [8].

The advantage of using antimicrobial packaging is the slow migration of the active compound to the product, as the active compound is gradually released from the package, allowing the activity to be extended during the storage phase of food distribution [9]. Gennadios et al. [10] showed other benefits of using films on meat including the reduction in moisture loss during storage of meat, keeping the juices of fresh meat and chicken, decreasing fat rancidity, and controlling the loss of flavor and absorption of foreign odors.

Bacteriocins incorporated into films can control bacteria by reducing the growth rate and/or prolonging the lag phase of microbes or by inactivating microbes by contact [11]. As an example of the best-studied bacteriocin, nisin, Coma et al. [12] succeeded in producing an antimicrobial film with nisin incorporated in hydroxypropyl methylcellulose solution, which was effective against *Listeria innocua* and *Staphylococcus aureus*. Broiler skin contamination with *Salmonella* typhimurium and spoilage microbes was reduced using nisin-activated packaging films [13] In addition to the examples for nisin, the poly(lactic acid)/sawdust particle biocomposite film impregnated with pediocin PA-1/AcH successfully inhibited approximately 99% of the total *Listeria* population on raw sliced pork during refrigerated storage [14]. Moreover, bacteriocin 7293, produced by *Weissella hellenica* BCC 7293, was diffusion-coated onto a biocomposite film of poly sawdust particles. The films produced successfully inhibited both gram-positive and gram-negative bacteria that commonly contaminate *Pangasius* fish fillets [15]. Plantaricins, bacteriocins produced by *Lactiplantibacillus plantarum*, are characterized by their stability at high temperatures and in acidic environments [16]. Plantaricin has been used as an active ingredient in food packaging films, resulting in a reduction in *L*. *monocytogenes* in chilled meat [17]. Furthermore, enterocins A and B were successfully utilized to control *L*. *innocua* in a variety of meat and its products [18]. The technology of the production of food packaging activated with bacteriocins is predicted to advance in the coming years, due to the demand for more natural foods [16].

Egyptian sources have shown great potential for discovering LAB bacteriocins. Notably, two bacteriocins with unique antimicrobial properties, plantaricin W and enterocin F4-9 were identified from Egyptian isolates, highlighting their potential for innovative food packaging applications [19]. These two bacteriocins can be considered good candidates for active agents in biopolymer film preparation. Plantaricin W (Plw) is a two-peptide bacteriocin produced by some strains of *L. plantarum*: the two peptides plantaricin Wα (Plwα; comprising 29 residues) and plantaricin Wβ (Plwβ; comprising 32 residues). Plw shows antimicrobial activity against a wide range of Gram-positive bacteria including *Lactobacillus* spp., *Oenococcus oeni*, *Leuconostoc mesenteroides*, *Pediococcus acidilactici*, *Pediococcus pentosaceus*, *Enterococcus faecalis*, *Bacillus subtilis*, *Listeria innocua*, *Listeria monocytogenes*, *Propionibacterium freudenreichii*, and *Staphylococcus aureus* [20]. Enterocin F4-9 is a novel *O*-linked glycosylated bacteriocin consisting of 47 amino acid residues, which shows antimicrobial activity even against a Gram-negative strain, *Escherichia coli* JM109, as well as Gram-positive bacteria [21].

To the best of our knowledge, this is the first report on the use of plantaricin W and enterocin F4-9 for food packaging applications. Combining the two bacteriocins has the potential to provide broader antimicrobial protection, particularly against Gram-negative bacteria that are tolerant to most LAB bacteriocins. Previous research has focused on incorporating a single bacteriocin, such as nisin or pediocin, into a food packaging system. In this study, we presented the application of plantaricin W and enterocin F4-9 to control the undesirable bacteria in meat through active food packaging.

## 2. Results

### 2.1. Antilisterial Activity of CMC Film Activated with Plantaricin W

**Figure 1:**
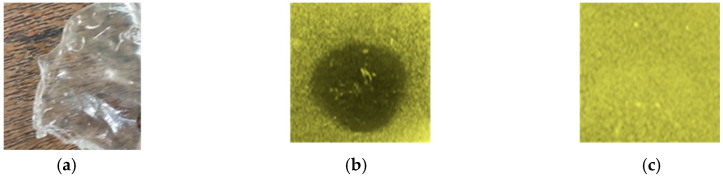
Antimicrobial activity of the activated film with plantaricin W. The produced CMC film (**a**), antimicrobial activity of the activated film with plantaricin W (**b**), and CMC film without plantarcin W (**c**). The antimicrobial property was assessed by the ability to inhibit the growth of *L*. *monocytogenes* ATCC BAA-679. As indicated, the activated film exhibited antimicrobial activity, but the untreated one exhibited no antimicrobial activity.

The film incorporated with plantaricin W was active against *L. monocytogenes* ATCC BAA-679 in the agar plate assay and yielded a clear, homogenous inhibition zone, suggesting that the bacteriocin was uniformly bound to the surface of the film and diffused regularly into the agar. However, the untreated film did not show any antilisterial activity, as shown in Figure 1.

### 2.2. Antimicrobial Activity of CMC Film Incorporated with Plantaricin W and Enterocin F4-9 Against Meat Microflora

**Figure 2:**
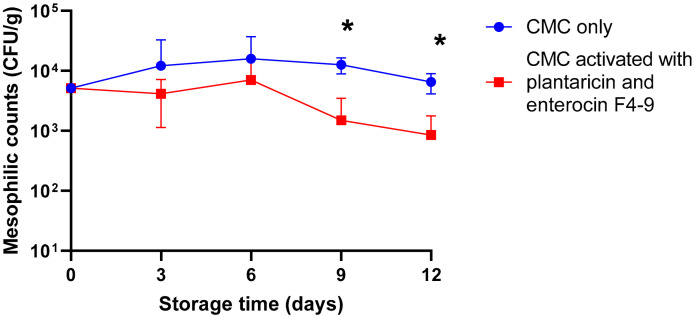
Mesophilic counts of beef minced meat. Meat samples were incubated for 12 days at 4 °C, packed with CMC only, and CMC activated with plantaricin W and enterocin F4-9 films. The Error bars represent the standard deviations derived from triplicate experiments. An asterisk indicates a statistically significant difference (*p* < 0.05) between counts of control and treated samples at the same time of storage.

To study the consequences of incorporating plantaricin W and enterocin F4-9 into CMC film to improve the shelf life of beef minced meat. The mesophilic aerobic bacteria counts were measured in samples of the control and those treated with bacteriocins that were stored for 12 days under refrigerated conditions. The mesophilic counts of samples treated with bacteriocins after 9 and 12 days of incubation were significantly lower than the control one by about 1 log, with a reduction rate of 86.67% on day 12 (Table 1), due to the inhibitory effect of plantaricin W and enterocin F4-9 (Figure 2).

**Figure 3:**
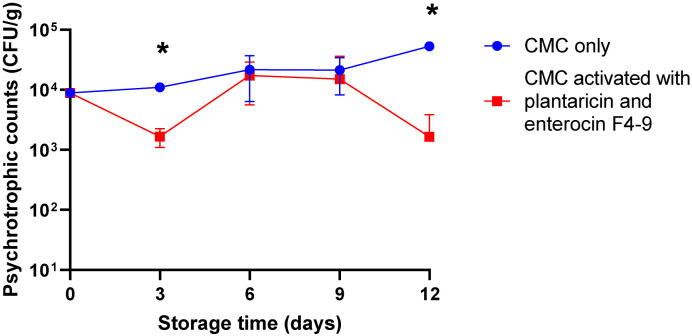
Psychrotrophic counts of beef minced meat. Meat samples were incubated for 12 days at 4 °C, packed with CMC only, and CMC activated with plantaricin W and enterocin F4-9 films. The Error bars represent the standard deviations derived from triplicate experiments. An asterisk indicates a statistically significant difference (*p* < 0.05) between counts of control and treated samples at the same time of storage.

Furthermore, after 12 days of storage, the control samples showed an increase in the number of psychrotrophic bacteria of 3 × 10^4^ CFU/g, whereas the samples treated with the active film were significantly reduced with counts of 1 × 10^3^ CFU/g (Figure 3), with a reduction rate of 96.67% after 12 days (Table 1).

### 2.3. Effect of CMC Film Incorporated with Plantaricin W and Enterocin F4-9 on the pH of Meat

**Figure 4:**
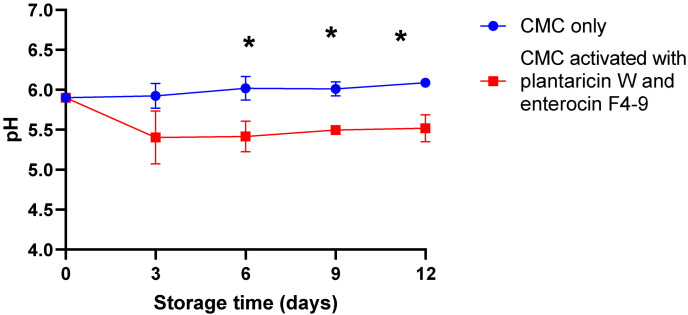
pH values of beef minced meat. Meat samples were incubated for 12 days at 4 °C, packed with CMC only, and CMC activated with plantaricin W and enterocin F4-9 films. The Error bars represent the standard deviations derived from triplicate experiments. An asterisk indicates a statistically significant difference (*p* < 0.05) between counts of control and treated samples at the same time of storage.

The pH is an important physicochemical parameter of food quality. The pH of the meat may affect the color and tenderness of the meat [22]. In this study, the initial pH of meat samples was 5.9, which, after treatment with bacteriocins, remained at 5.5 after 12 days of storage. However, the pH of the untreated samples increased to 6.1. In contrast, the treated meat samples exhibited significantly lower pH values at 6, 9, and 12 days of storage compared to the control group (Figure 4).

### 2.4. Effect of CMC Film Incorporated with Plantaricin W on Listeria During the Storage of Beef Minced Meat

**Figure 5:**
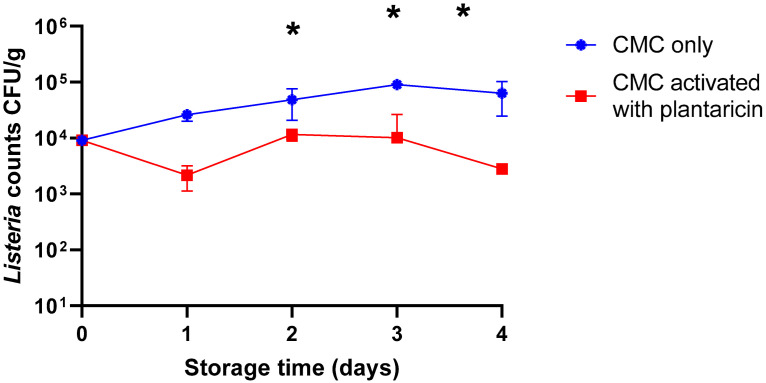
Evaluation of the antilisterial activity of active food packaging in minced beef meat stored for 4 days at refrigeration temperature (4 ± 1 °C). Contaminated meat with approximately 10^4^ CFU/g of *Listeria monocytogenes* ATCC BAA-679 was incubated with CMC only, and CMC was activated with plantaricin W films. The Error bars represent the standard deviations derived from triplicate experiments. An asterisk indicates a statistically significant difference (*p* < 0.05) between counts of control and treated samples at the same time of storage.

The developed antimicrobial films were also used in the challenge tests of the storage of meat products artificially contaminated by *L. monocytogenes* ATCC BAA-679. The result of the viable counts of *Listeria* on beef minced meat at different times of storage at 4 °C is shown in Figure 5. The treatment of beef mince samples with CMC film incorporating plantaricin W significantly reduced the *Listeria* population by approximately 1 log cycle from day 2 to day 4, achieving a 98.33% reduction rate by day 4. Furthermore, the reduction rates of mesophilic and psychrotrophic bacteria were 86.67% and 96.67%, after 12 days of storage, respectively (Table 1).

## 3. Discussion

Bacteriocins are natural antimicrobial peptides that are generally recognized as safe compounds, and their utilization in food packaging does not pose a health risk to consumers [23]. Incorporating bacteriocins into food packaging offers several advantages, including the effective control of food-borne pathogens and the extension of food shelf life. This aligns with consumers’ demand for natural food preservatives. In this report, we have described the usefulness of the active food package for controlling the undesirable bacteria in meat. Plantaricin W is a two-peptide bacteriocin that can inhibit various gram-positive bacteria [20]. Plantaricin W showed activity against a major food-borne pathogen, *L. monocytogenes* ATCC BAA-679.

Bacteriocins have various antimicrobial mechanisms, forming pores in the bacterial membrane, disrupting the membrane potential, inhibiting cell wall biosynthesis, targeting specific membrane proteins or transport systems, and inducing cell lysis [24]. Several researchers reported the efficacy of antimicrobial films activated by nisin for controlling food microbes [12,25,26]. CMC has a lot of advantages, such as being easily dissolved in cold water, maintaining elasticity, being hygroscopic, and absorbing moisture released by packaged food [27]. Furthermore, CMC shows promise due to its film-forming properties, which include mechanical strength, barrier qualities, translucency, and thermal resistance [28]. Adding active ingredients to CMC-based packaging, including antimicrobials and antioxidants, provides a unique benefit. These bioactive compounds offer long-term defense against oxidation and spoilage since they can be released gradually [29]. Based on our knowledge, this is the first report about the application of plantaricin W in meat. The developed active CMC films were assayed for their antimicrobial activity against *L. monocytogenes* ATCC BAA-679 in a challenge test of the storage of beef minced meat at refrigeration temperature. A significant reduction in the *Listeria* population was obtained from the second day until the end of the experiment in samples packed with active CMC film with plantaricin W compared with the control (Figure 5). Ming et al. [30] investigated the antilisterial activity of cellulosic casing sprayed with pediocin AcH and recorded that the film prevented *listeria* growth in meat samples during 12 weeks of storage at 4 °C. The bacteriocin produced by *Lactobacillus* (*Latilactobacillus*) *curvatus* 32Y was incorporated into polyethylene films. The produced film significantly reduced the count of *Listeria monocytogenes* in hamburger samples during the first 24 h of storage, and the *Listeria* population remained lower than the samples packed with untreated film [7]. Normally, raw meats are contaminated with a moderate level of *L. monocytogenes*, not more than 100 CFU/g [31]. We suggested that the produced film in this study is an effective approach to reducing the population of *Listeria* on meat during refrigerated storage.

Another antimicrobial food packaging was activated by our novel bacteriocin (enterocin F4-9) and plantaricin W. The combination of plantarcin W and enterocin F4-9 has the potential to broaden the antimicrobial activity of food packaging, particularly against Gram-negative bacteria, through complementary mechanisms. This broader activity is hypothesized to result from the unique structure of enterocin F4-9, which includes *O*-linked *N*-acetylglucosamine [32]. The synthesis of glycocin derivatives lacking sugar moieties revealed a significant reduction in antimicrobial activity against *E*. *coli* D22, highlighting the importance of glycosylation in mediating interactions with Gram-negative membranes [33]. The mesophilic aerobic counts were measured in treated and untreated samples stored for 12 days under refrigeration (Figure 2). The mesophilic count in treated samples was kept lower than in samples packed with untreated film. There was a significant reduction in total mesophilic count between the treated and control samples at 9 and 12 days of the experiment, which will extend the shelf life of the treated samples. Reductions in total mesophilic counts when meat samples were treated with active food packaging were previously reported [34,35,36]. Guerra et al. [35] applied cellophane incorporated with nisin in meat, where the mesophilic counts in treated samples were 7.9 × 10^3^ CFU/g and significantly lower than the control sample, suggesting the effect of nisin would extend the shelf life of meat.

Enterocin F4-9 and plantaricin W were successfully able to keep the psychrotrophic bacterial population in treated meat samples lower than the sample packed with untreated film (Figure 3). Briefly, the release of the active substance from the film is responsible for the initial decrease in the psychotropic bacterial count in treated samples by active film compared to control at 3 days. However, at 6 and 9 days, we observed a slight increase in the psychotropic bacterial count, which may be explained by some bacteria adapting to the presence of the active film. However, at 12 days, the bacterial count returns to decrease in comparison to the control because of the continuous release of active substances from the film. Fangio and Fritz [34] reported that meat treated with a bacteriocin-like substance produced by *Bacillus cereus* P9 became spoiled between 9 and 12 days, while the untreated samples became spoiled after 3 and 6 days of storage. Similar observations were also recorded by Fiorentini et al. [37]. The overall impact on psychrotrophic and mesophilic bacterial populations, which encompass a range of Gram-negative species including *E*. *coli* and common meat-spoiling organisms, suggests that the bacteriocins exert an inhibitory effect on the Gram-negative bacteria present in the meat matrix.

pH is an indicator of meat freshness. Acceptable meat has a pH of around 5.8 after 24 h of slaughter [38]. In this study, the initial pH was 5.90, which reached 5.52 after 12 days of storage due to the diffusion of bacteriocins to the meat, while it increased to 6.09 for the untreated sample (Figure 4). Fiorentini et al. [37] had an initial meat pH of 6.07 that decreased to 5.81 after the addition of cell-free bacteriocinogenic supernatants, while it reached 6.08 for the control.

In the current work, a new approach to prolonging the shelf life of beef by combining several bacteriocins was demonstrated. The results obtained, as demonstrated in Table 1, emphasize that biopreservation with LAB bacteriocins is a promising way to combat food microbes and suggest the potential application of bacteriocin-active films in meat preservation. Sensory evaluation showed that there was no change in color and odor, which reflected the degree of lipid and protein deterioration.

This study is notable for its integration of two distinct bacteriocins, plantaricin W and enterocin F4-9, into CMC films to broaden the antimicrobial spectrum. The use of these proteinaceous bacteriocins, which are generally recognized as safe, represents a promising strategy for developing active food packaging. Furthermore, the produced film demonstrated its efficacy in a real food matrix, unlike some earlier studies, which primarily reported results from in vitro assays rather than actual food products. Although CMC is well recognized for its excellent film-forming properties, ongoing studies are currently underway to further characterize the produced films and to include Gram-negative bacterial challenge data.

## 4. Materials and Methods

### 4.1. Bacterial Strains and Media

Strain A4-6 was isolated from Egyptian yogurt and identified as *Lactiplantibacillus plantarum* by the 16S rRNA gene sequence. LC/MS was used to identify the bacteriocin produced by *L. plantarum* A4-6 as described by Zendo et al. [5], and it was identified as plantaricin W. *L. plantarum* A4-6 and *Enterococcus faecalis* F4-9 were stored at −80°C in MRS medium (De Man Rogosa Sharpe, Oxoid, Basingstoke, UK) with 15% glycerol. Before use, *L*. *plantarum* A4-6 and *Enterococcus faecalis* F4-9 were propagated in an MRS medium at 30 °C. *Listeria monocytogenes* ATCC BAA-679 and *Heyndrickxia coagulans* JCM 2257^T^ were propagated in Tryptic Soy Broth (BD, Sparks, MD, USA) supplemented with 0.6% yeast extract (Nacalai Tesque, Kyoto, Japan) (TSBYE) at 37 °C, while *Enterococcus faecalis* JCM 5803^T^ was cultivated in MRS medium at 37 °C.

### 4.2. Purification of Plantaricin W and Enterocin F4-9

Partial purification of plantaricin W was performed based on the method described by Holo et al. [20] with minor modifications. *L*. *plantarum* A4-6 was cultivated in MRS medium at 30 °C. The cells were removed by centrifugation at 6000× *g* for 15 min, and 25 g of activated Amberlite XAD-16 resin (Sigma-Aldrich, St. Louis, MO, USA) was mixed with the culture supernatant and slowly shaken overnight at 4 °C. The resin matrix was washed with 50 mL of Milli-Q water and 500 mL of 40% (*v*/*v*) ethanol. Plantaricin W was eluted with 150 mL of 70% (*v*/*v*) 2-propanol containing 0.1% trifluoroacetic acid (TFA, Nacalai Tesque). To remove the 2-propanol, the eluted active fraction was evaporated. The eluted substance was concentrated using freeze-drying. The powder was referred to as partially purified plantaricin W and was stored at −30 °C. The antibacterial activity of the culture supernatant and partially purified plantaricin W was determined by using the spot-on-lawn method against *H. coagulans* JCM 2257^T^ as the indicator strain. Briefly, 10 µL of a two-fold fs of a bacteriocin preparation was spotted onto a double layer comprised of 5 mL of TSBYE with 1% agar and inoculated with an overnight culture of an indicator strain as the upper layer and 10 mL of MRS medium with 1.2% agar as a bottom layer. After incubating overnight at the appropriate temperature for the indicator strains, the bacterial lawns were checked for inhibition zones [39]. Enterocin F4-9 was partially purified from 1 L of the culture supernatant of *E. faecalis* F4-9 cultivated in MRS medium by Amberlite XAD-16 resin (Sigma-Aldrich, St. Louis, MO, USA), enterocin F4-9 was eluted with 150 mL of 70% (*v*/*v*) 2-propanol containing 0.1% trifluoroacetic acid (TFA, Nacalai Tesque) as described by Maky et al. [21]. Then, the eluted substance was concentrated using freeze-drying. The powder was referred to as partially purified enterocin F4-9 and was stored at −30 °C.

### 4.3. Production of Two Kinds of Active Films

CMC films were produced by modification of the method used by Chana-Thaworn et al. [40]. Briefly, 0.2 g of CMC was dissolved in 20 mL of distilled water and shaken for 10 min using a vortex at medium intensity. Then, 0.5 g of sorbitol (Sigma-Aldrich, St. Louis, MO, USA) was added to improve the flexibility, continuity, cohesiveness, and adhesiveness of the packaging materials. Finally, partially purified plantaricin W (the total activity in the culture supernatant was previously determined to be 6.400 × 10^3^ AU/mL [19]) was added at a concentration of 0.02% (*w*/*v*) and stirred for 5 min using the vortex at medium intensity, and then the mixture was degassed under a vacuum and poured onto flat plates. The plates were subjected to a dehydration process to prevent mold growth by holding at 55 °C for 4–6 h and then cooled to room temperature before peeling the films off the plates. Another CMC film was produced as described previously, with the addition of 0.02% (*w*/*v*) each of enterocin F4-9 (the total activity in the culture supernatant was previously determined to be 2.00 × 10^5^ AU/mL [21]) and plantaricin W [40,41].

### 4.4. Antilisterial Activity of the Plantaricin W-Activated CMC Films

Antilisterial activity of CMC film activated by plantaricin W was assayed using agar diffusion assay [41]. Briefly, a circle of small diameters of the produced film was situated on the surface of Tryptic Soy Agar (BD, Sparks, MD, USA) supplemented with 0.6% yeast extract, inoculated with 1% of an overnight culture of *L. monocytogenes* ATCC BAA-679. In addition, untreated films were also assayed as a negative control. The plate was incubated at 37 °C for 24 h and the antagonistic activity was evaluated by observing a clear zone of growth inhibition around the tested film.

### 4.5. Activity of CMC Films Incorporated with Plantaricin W and Enterocin F4-9 During the Storage of Beef Minced Meat

The antimicrobial strength of the developed CMC film incorporated with plantaricin W and enterocin F4-9 was evaluated to control the meat microflora during the storage of minced beef. Samples of minced beef were packed in direct contact with treated films. The samples packed with untreated films served as a negative control. Each sample was placed in a sterile plastic bag and kept under refrigeration (4 ± 1 °C). Each treatment was made in triplicate. Microbiological counts for mesophilic bacteria and psychrotrophic aerobic bacteria of the samples were performed at different time intervals (0, 3, 6, 9, and 12 days) by the pouring plate technique. Briefly, on days 0, 3, 6, 9, and 12, samples were homogenized in 0.1% peptone water (BD, Sparks, MD, USA) at a 1:10 ratio. Furthermore, decimal dilutions were made using the same diluents. An aliquot of each dilution was plated on Plate Count Agar (BD, Sparks, MD, USA) for determining the growth of mesophilic aerobic bacteria count; the plates were incubated at 37 °C for 2 days. For the psychrotrophic aerobic bacteria count, the same procedures were conducted with incubation of the plates at 20 °C for 5 days. Acceptable mesophilic counts for minced beef are generally considered to be less than 10^6^ CFU/g, as recommended by the International Commission on Microbiological Specifications for Foods (ICMSF) [42]. Furthermore, the pHs for the control and treated samples were measured. Briefly, 1 g of each sample was mixed with 10 mL of distilled water, and the pH was measured using a pH meter HM-25R (DKK-TOA, Tokyo, Japan) on days 0, 3, 6, 9, and 12.

### 4.6. Antilisterial Activity of CMC Film Incorporated with Plantaricin W During the Storage of Beef Minced Meat

The antilisterial strength of the developed CMC film incorporated with plantaricin W was evaluated for control of *L. monocytogenes* ATCC BAA-679 during storage of minced meat. According to Woraprayote et al. [14], with some modifications, samples of minced beef were sterilized with UV light for 15 min on each side. Sterilized meat samples were then inoculated with 1 μL of an overnight culture of *L. monocytogenes* ATCC BAA-679 to obtain a final *Listeria* density on meat samples of around 10^4^ CFU/g. Then, samples were packed in direct contact with treated and untreated films and stored at 4 °C. After 0, 1, 2, 3, and 4 days of storage, viable counts of *Listeria* on Tryptic soy agar supplemented with 0.6% yeast extract were performed by making a ten-fold dilution in 0.1% peptone water (BD, Sparks, MD, USA). The experiments were performed in a triplicate manner, and the results were expressed as CFU/g.

### 4.7. Statistical Analysis

Three experimental replicates were conducted each day. Statistical analysis of the results was conducted by analysis of variance (ANOVA) using SPSS 16.0. Significance was defined at a level of *p* < 0.05.

## Figures and Tables

**Table 1:** An overview of the effect of CMC film application on the microbial population (mean ± SD CFU/g) of beef mince during cold storage.

	Mesophilic Bacteria					Psychrotrophic Bacteria					*Listeria monocytogenes*				
Day	0	3	6	9	12	0	3	6	9	12	0	1	2	3	4
Inactive film	5 × 10^3^ ± 2 × 10^3^	1 × 10^4^ ± 2 × 10^4^	1 × 10^4^ ± 2 × 10^4^	1 × 10^4^ ± 3 × 10^3^	6 × 10^3^ ± 2 × 10^3^	8 × 10^3^ ± 7 × 10^3^	1 × 10^4^ ± 1 × 10^3^	2 × 10^4^ ± 1 × 10^4^	1 × 10^4^ ± 1 × 10^4^	3 × 10^4^ ± 1 × 10^4^	9 × 10^3^ ± 3 × 10^2^	2 × 10^4^ ± 9 × 10^3^	4 × 10^4^ ± 2 × 10^4^	9 × 10^4^ ± 1 × 10^4^	6 × 10^4^ ± 3 × 10^4^
Active film	5 × 10^3^ ± 2 × 10^3^	4 × 10^3^ ± 3 × 10^3^	7 × 10^3^ ± 9 × 10^3^	1 × 10^3^ ± 1 × 10^3^	8 × 10^2^ ± 9 × 10^2^	8 × 10^3^ ± 7 × 10^3^	1 × 10^3^ ± 6 × 10^2^	1 × 10^4^ ± 1 × 10^4^	1 × 10^4^ ± 2 × 10^4^	1 × 10^3^ ± 2 × 10^3^	9 × 10^3^ ± 3 × 10^2^	2 × 10^3^ ± 1 × 10^3^	1 × 10^4^ ± 8 × 10^3^	1 × 10^4^ ± 1 × 10^4^	1 × 10^3^ ± 1 × 10^3^
Reduction% ^a^	0	60	30	90	86.67	0	90	50	0	96.67	0	90	75	88.89	98.33

^a^: Reduction rate (%) = [(mean count of inactive film − mean count of active film)/mean count of inative film] × 100

## Data Availability

The data presented in this study are available on request from the corresponding author.
